# Estimation of item parameters and examinees’ mastery probability in each domain of the Korean Medical Licensing Examination using a deterministic inputs, noisy “and” gate (DINA) model

**DOI:** 10.3352/jeehp.2020.17.35

**Published:** 2020-11-17

**Authors:** Younyoung Choi, Dong Gi Seo

**Affiliations:** 1 Department of Adolescent Coaching Counseling, Hanyang Cyber University, Seoul, Korea; 2 Department of Psychology in College of Social Science & Hallym Applied Psychology Institute, Hallym University, Chuncheon, Korea

**Keywords:** Diagnostics classification model, DINA model, Large-scale assessment, Classification and learning

## Abstract

**Purpose:**

The deterministic inputs, noisy “and” gate (DINA) model is a promising statistical method for providing useful diagnostic information about students’ level of achievement, as educators often want to receive diagnostic information on how examinees did on each content strand, which is referred to as a diagnostic profile. The purpose of this paper was to classify examinees of the Korean Medical Licensing Examination (KMLE) in different content domains using the DINA model.

**Methods:**

This paper analyzed data from the KMLE, with 360 items and 3,259 examinees. An application study was conducted to estimate examinees’ parameters and item characteristics. The guessing and slipping parameters of each item were estimated, and statistical analysis was conducted using the DINA model.

**Results:**

The output table shows examples of some items that can be used to check item quality. The probabilities of mastery of each content domain were also estimated, indicating the mastery profile of each examinee. The classification accuracy and consistency for 8 content domains ranged from 0.849 to 0.972 and from 0.839 to 0.994, respectively. As a result, the classification reliability of the diagnostic classification model was very high for the 8 content domains of the KMLE.

**Conclusion:**

This mastery profile can provide useful diagnostic information for each examinee in terms of each content domain of the KMLE. Individual mastery profiles allow educators and examinees to understand which domain(s) should be improved in order to master all domains in the KMLE. In addition, all items showed reasonable results in terms of item parameters.

## Introduction

### Background/rationale

Assessments and psychometric models developed under the trait psychology perspective have been reliable methods for evaluating the general state of students’ knowledge, skills, and abilities when the purpose of measurement is to compare students’ abilities and to select students who have developed mastery in the context of a licensing examination. However, overall scores of this type do not offer sufficient useful information for the purposes of (1) measuring multidimensional contextual knowledge, skills, and abilities; (2) measuring complicated tasks reflecting complex knowledge, skills, and abilities; (3) understanding distinguishable systematic patterns associated with different characteristics of groups; and (4) providing diagnostic information connected with the curriculum and instruction. For these purposes, it is necessary to obtain more information from assessment results through various measurement models.

More specifically, the main purpose of a large-scale assessment is to compare students’ achievement levels and to make pass/fail decisions based on general proficiency. This is usually done by students’ overall scores, which are used to assign students to specific performance levels. However, this information has very little instructional usefulness in terms of what should be done to improve individual students’ levels of achievement. That is, overall test scores from large-scale assessments offer relatively little diagnostic information about a student’s strengths and weaknesses [1]. Diagnostic information is more informative for students, instructors, and assessment/instruction developers from the perspective of learning and improving the quality of assessments and the curriculum [2]. In light of these issues, diagnostic classification models (DCMs) have been proposed as psychometric models.

DCMs are statistical models that were originally developed to classify students in terms of their mastery status for each attribute [3,4]. DCMs contain multiple attributes, which refer to latent aspects of knowledge, skills, and abilities that are supposed to be measured in an assessment. Students’ mastery status for the attributes of interest are estimated based on their observed response patterns. A composite of a student’s mastery statuses for the attributes is referred to as an attribute profile. Therefore, the attribute profile is a pattern used for providing diagnostic feedback. Several DCMs have been proposed, such as deterministic inputs, noisy “and” gate (DINA), deterministic inputs, noisy “or” gate (DINO), and the re-parameterized unified model.

These models differ depending on the variables of interest and the condensation rules that are used for modeling attributes; however, a central concept of modeling is linking the diagnostic classification with cognitive psychological findings [4]. Since multiple attributes are involved and tasks can depend on more than one attribute, their relationships are represented by a complex loading structure, often called a Q matrix [4]. A Q matrix contains the targeted attributes and specification of which attributes are measured by which task(s) based on substantive theoretical input (e.g., a domain specialist for the relevant examination). To construct a Q matrix, many sources may be used, such as subject matter experts’ opinions, cognitive developmental theory, learning science, and learning objectives in the curriculum.

Educators often want diagnostic information from assessments, and in particular, educators in the health professions often want to provide feedback to a given student based on how he or she does on each content strand. However, most assessments are developed to provide only a single total score [3]. Most score reports in the health professions provide a total score or pass-fail decisions based on classical test theory. DCMs are psychometric models that characterize examinees’ responses to test items through the use of categorical latent variables that represent their knowledge [5]. Thus, DCMs have become a popular area of psychometric research. However, few application studies using DCMs with health professions data have been reported [6]. The purpose of this study was to conduct a DINA analysis using Korea Health Professional Licensing Examination data in order to provide diagnostic information about each content domain in this licensing examination.

### Objectives

The purpose of this study was to conduct a DINA analysis using Korean Medical Licensing Examination (KMLE) data in order to provide diagnostic information about each content domain in this licensing examination. Specifically, the guessing and slipping parameters of items and the mastery probabilities of examinees in each domain were estimated. The ultimate objective of this study was to evaluate the classification reliability of mastery in 8 content domains by using DINA and investigate the application of DCM in KMLE.

## Methods

### Ethics statement

The raw data file was obtained from the Korea Health Professional Licensing Examination Institute for research purposes. The open data source does not contain any identification or personal information about the examinees. Therefore, informed consent and the requirement for institutional review board approval were exempted according to the Enforcement Rule of the Bioethics and Safety Act under Korean law.

### Study design

This was a diagnostic analysis of high-stakes exam results based on a DCM for identifying item parameters and examinees’ mastery status.

### Data sources/measurement:

Data from the KMLE in 2017, including 360 items and 3,259 examinees, were analyzed in this study. The data are available from https://doi.org/10.7910/DVN/PETWZF (Dataset 1) [7]. The 8 content domains of the KMLE are described in Table 1.

### Q matrix

For a DCM analysis, it is necessary to create a Q matrix, which provides information about the relationship between items and content domains. Since this study analyzed 360 items, Table 2 shows the Q matrix with a few examples of item information. The entire Q matrix is available in Dataset 2.

### Statistical methods

The following DINA model was used in this study:

P(Xij=1ηij)=gi(1-ηij)(1-si)ηij ,

where *x_ij_* denotes the response of examine *j* to item *i* (where *i*=1,…,*i*), with 1 and 0 representing a correct or incorrect response, respectively, and *g_i_* and *s_i_* representing the guessing and slipping parameters for the item *i*, respectively. Additionally, *η_ij_* is a binary indicator related to the Q matrix, and the following equation indicates whether examinee *j* has mastered all attributes required by item *i*:

$$\eta_{ij}=\prod_{k=1}^{K}{\left(\alpha_{jk}\right)}^{q_{jk}}$$

The parameter *α_ik_* refers to classification *k* in latent class *j*, which is either 1 or 0 for *k*. The parameter *q_ik_* refers to the entry in row *i*, column *k* of the *Q* matrix, an attributes-to-items mapping with dimensions *I* × *K* for which individual entries take values according to

$$q_{ik}=\left\{\begin{array}{l} 1\;\mathrm{if}\;\mathrm{item}\;i\;\mathrm{requires}\;\mathrm{attirbute}\;k\\ 0\;\mathrm{otherwise} \end{array}\right.$$

The general model for DCMs is as follows [5]:

$$P(X_{r}=x_{r})=\sum_{C}\;\pi_{c}\prod_{I}\;p_{ic}^{x_{ir}}{\left(1-p_{ic}\right)}^{1-x_{ir}}$$

Different DCMs provide different parameterizations of P_c_ based on the relation between tasks and attributes, and among attributions. A unified model referred to as the log-linear cognitive diagnosis model (LCDM) framework can capture different DCMs, such as DINA, DINO, and re-parameterized unified model [8]. The LCDM is as follows:

P(Yij=1αi,qj)=exp (λ0j+λjTh(αi,qj))1+exp (λ0j+λjTh(αi,qj))

where *i* and *j* denote the student and task, respectively; *λ*_0*j*_ is an intercept and *λ_j_* represents a vector of the coefficient indicating the effects of attribute mastery on the response probability for item *j*; and *h*(*α_i_*, *q_j_*) is a set of linear combinations of *α_i_* and *q_j_*. The intercept can be interpreted as the guessing parameter, the *λ_ju_* parameters represent the main effects of each attribute *u* on the response probability for item *j*, and the *λ_juv_* parameters represent the 2-way interaction effects of the combination of the mastery status for attributes *u* and *v* on the response probability for item *j*. We used the CDM R package (The R Foundation for Statistical Computing, Vienna, Austria; https://cran.r-project.org/) to implement the DINA model [9]. The main code to implement the DINA model is as follows:

install.packages(“CDM”)
library(CDM)
dinadat <- read.table( "data2017.csv", header = F,sep=",")
qmatrix <- read.table( "qmatrix.csv", header=F, sep="," )
d1 <- CDM::din( dinadat, q.matrix=qmatrix)
summary(d1)

## Results

### Model fit

For the DINA model in this dataset, the Akaike information criterion and Bayesian information criterion were 998,956 and 1,004,893, respectively. The mean root mean square error of approximation for item fit was 0.045, indicating that this model fit the data well [10].

### Estimation of the guessing and slipping parameters of items

Some examples of the guessing and slipping parameters are shown in Table 3. (All guessing and slipping parameters are available in Dataset 3.)

The guessing parameter indicates the probability of a correct response to an item that a respondent should answer incorrectly. In this context, the idea that the respondent should answer the item incorrectly means that the respondent has non-mastery of at least 1 required content domain. The slipping parameter represents the probability of an incorrect response to an item that a respondent should answer correctly because the respondent has mastery of all required content domains. For example, the probability of a correct response to item 1 using guessing was 0.008, while the probability of an incorrect response to item 1 for high-ability students was 0.984. Thus, item 1 showed high discrimination. In contrast, for item 2, the guessing parameter was 0.962 and the slipping parameters was 0.016. Thus, item 2 showed very low discrimination. The average values of the guessing and slipping parameters in this exam were 0.647 and 0.228, respectively.

### Estimation of mastery probabilities of the examinees in each domain

This study estimated the mastery probability of each content domain for all examinees. Table 4 shows the probability of having mastery in each content domain for 3,259 examinees and the average probability of having mastery in each content domain. The full dataset on the estimation of mastery probabilities is available in Dataset 4. For example, the probabilities of having mastery in each content domain for examinee 2939 were 0.999, 0.999, 0.995,0 .899, 1, 0.965, 0.995, and 0.860, respectively, which means that this student had high probabilities of mastery in all content domains. In contrast, the corresponding probabilities for examinee 509 were 0.112, 0.135, 0.007, 0.053, 0.004, 0.002, 0.166, and 0.008, meaning that student 509 had low probabilities of mastery for all content domains. Thus, the mastery information from the DINA model provides precise predictions and diagnostic information for each content domain.

### Classification reliability of the diagnostic classification model

The classification accuracy and consistency of the DCM are described in Table 5. The item parameters and the probability distribution of latent classes using a simulation were compared with the classification of the real data. The classification accuracy and consistency of mastery were estimated using maximum a posterior classification estimator [11]. Classification accuracy and consistency were estimated for the separate classification of 8 content domains. Table 5 showed that classification accuracy of mastery for the 8 content domains ranged from 0.849 to 0.972 and the classification consistency ranged from 0.839 to 0.994. As a result, the classification reliability of the DCM was very high for the 8 content domains of the KMLE.

Students’ mastery probabilities for content domains 1–8 were 61.7%, 61.8%, 59.2%, 55.7%, 59.0%, 59.7%, 69.5%, and 42.4%, respectively.

## Discussion

### Key results

DCMs have become a popular area of psychometric research in educational testing. DCMs are psychometric models that classify students based on categorical latent variables related to the purpose of the assessment. This psychometric model is a promising means for students, instructors, and test developers to obtain more detailed diagnostic information [5]. This study conducted an analysis using the DINA model, which is one of the main types of DCMs, using response data from the KMLE. The output showed the guessing and slipping parameters, enabling the quality of items to be checked. In addition, the examinees’ mastery probabilities for each content domain were estimated. The mastery profile provides individual information on which domain(s) should be improved in order to master all domains in the KMLE. Therefore, educators can identify specific content domains for each examinee to improve their weaknesses in content domains. Finally, this study demonstrated that the subscore classification accuracy for mastery was very high, and subscores were reported consistently for all content domains, which means that the classification accuracy was very reliable based on the outcomes of the DCM.

### Interpretation

By investigating the guessing and slipping parameters for each item, test developers can check whether items are appropriate to measure examinees’ mastery of each content domain. Through the probabilities of mastery for all content domains, the DINA model provides precise predictions of diagnostic information in terms of each content domain for all examinees. In addition, the high classification accuracy and consistency estimated from the DINA model demonstrated that the KMLE has a high classification reliability for mastery of the 8 content domains.

### Limitation

The DINA was applied to a simple structural model, in which an item belongs to 1 content domain, whereas a complex structure model would allow each item to be assigned to multiple content domains and therefore have item loading to more than one content domain. The DINA model can also be applied to generate estimations for a complex structural model. Compensation and non-compensation rules that can take into account the complexity of relationships between items and content domains have been proposed for DCMs. Since limited content information is available about the KMLE, this study only applied a simple structural model. Therefore, a more complex structural model (i.e., with possible item loading to multiple domains) using DCMs would be a topic for future research. This study used only 1 type of DCM (the DINA model), and further research could examine several types of DCMs for medical education assessment.

### Conclusion

Despite the limitations of the current study, the mastery information in terms of subscores for each domain can be used for remediation to improve a student’s achievement. Such fine-grained information would be useful for competency-based education and formative purposes in professional activities. In addition, providing examinees detailed feedback from a DCM analysis can contribute to health professions education by identifying areas of weakness for improvement and enhancing students’ learning.

## Figures and Tables

**Table 1.** Item information in terms of content domains

Content domains	No. of items (%)
C1	45 (12.5)
C2	45 (12.5)
C3	45 (12.5)
C4	25 (6.94)
C5	154 (42.78)
C6	20 (5.56)
C7	20 (5.56)
C8	6 (1.67)
Total	360 (100)

**Table 2.** Q matrix for the Korean Medical Licensing Examination

Item	Content Domain C1	Content Domain C2	Content Domain C3	Content Domain C4	Content Domain C5	Content Domain C6	Content Domain C7	Content Domain C8
1	0	1						0
2	0	1						0
3	0	1						0
4	1	0						
…								
356	0	1						0
357	0	0			1			0
358	0	0		1				0
359	0	0			1			0
360	0	0		1				0

**Table 3.** Guessing and slipping parameters of each item

Item ID	Guessing	Slipping
1	0.008	0.984
2	0.962	0.016
3	0.191	0.796
4	0.473	0.343
5	0.903	0.036
6	0.801	0.075
7	0.234	0.509
8	0.076	0.848
9	0.906	0.035
10	0.736	0.080
…		
356	0.867	0.085
357	0.709	0.052
358	0.808	0.048
359	0.647	0.094
360	0.418	0.409
Average	0.647	0.228

**Table 4.** Probability table of having mastery in each content domain for all examinees

examinee ID	Domain 1	Domain 2	Domain 3	Domain 4	Domain 5	Domain 6	Domain 7	Domain 8
2939	0.999	0.999	0.995	0.899	1.000	0.965	0.995	0.860
800	0.998	0.998	0.999	0.989	0.901	0.850	0.940	0.626
1689	1.000	0.997	0.997	0.838	1.000	0.886	0.996	0.820
1900	1.000	1.000	1.000	0.997	1.000	0.998	0.980	0.915
…								
1559	0.996	0.998	0.995	0.877	1.000	0.898	0.992	0.683
409	0.988	0.996	1.000	0.927	1.000	0.940	0.985	0.454
2625	0.992	0.998	0.998	0.995	1.000	0.989	0.984	0.859
1917	0.015	0.594	0.130	0.151	0.002	0.377	0.808	0.002
509	0.112	0.135	0.007	0.053	0.004	0.002	0.166	0.008
Average	0.617	0.618	0.592	0.557	0.590	0.597	0.695	0.423

**Table 5.** Classification accuracy and consistency for the deterministic inputs, noisy “and” gate (DINA) model

Content domains	Classification accuracy	Classification consistency
C1	0.932	0.931
C2	0.944	0.953
C3	0.939	0.945
C4	0.918	0.921
C5	0.972	0.994
C6	0.885	0.874
C7	0.890	0.876
C8	0.847	0.839

## Footnotes

**Data availability** Data files are available from Harvard Dataverse: https://doi.org/10.7910/DVN/X2DRTE Dataset 1. Raw data files of the Korea Medical Licensing Examination. Dataset 2. Q matrix data. Dataset 3. Item parameter data including slipping and guessing. Dataset 4. Examinees’ mastery profile data.

## Supplementary materials

Data files are available from Harvard Dataverse: https://doi.org/10.7910/DVN/X2DRTE

Supplement 1. Audio recording of the abstract.
